# Mutations in *HYAL2*, Encoding Hyaluronidase 2, Cause a Syndrome of Orofacial Clefting and Cor Triatriatum Sinister in Humans and Mice

**DOI:** 10.1371/journal.pgen.1006470

**Published:** 2017-01-12

**Authors:** Martina M. A. Muggenthaler, Biswajit Chowdhury, S. Naimul Hasan, Harold E. Cross, Brian Mark, Gaurav V. Harlalka, Michael A. Patton, Miho Ishida, Elijah R. Behr, Sanjay Sharma, Kenneth Zahka, Eissa Faqeih, Brian Blakley, Mike Jackson, Melissa Lees, Vernon Dolinsky, Leroy Cross, Philip Stanier, Claire Salter, Emma L. Baple, Fowzan S. Alkuraya, Andrew H. Crosby, Barbara Triggs-Raine, Barry A. Chioza

**Affiliations:** 1 RILD Wellcome Wolfson Centre, University of Exeter Medical School, Exeter, United Kingdom; 2 Department of Biochemistry & Medical Genetics, University of Manitoba, Winnipeg, Manitoba, Canada; 3 Department of Ophthalmology, University of Arizona College of Medicine, Tucson, Arizona, United States of America; 4 Department of Microbiology, University of Manitoba, Winnipeg, Manitoba, Canada; 5 Genetics Research Centre, St George’s University London, London, United Kingdom; 6 Genetics and Genomic Medicine, UCL Institute of Child Health, London, United Kingdom; 7 Cardiovascular Sciences Research Centre, St George's University of London, London, United Kingdom; 8 Pediatric Cardiology, Cleveland Clinic, Cleveland, Ohio, United States of America; 9 Department of Pediatric Subspecialties, Children’s Hospital, King Fahad Medical City, Riyadh, Saudi Arabia; 10 Department of Otolaryngology, University of Manitoba, Winnipeg, Manitoba, Canada; 11 Department of Small Animal and Materials Imaging Facility, University of Manitoba, Winnipeg, Manitoba, Canada; 12 Department of Clinical Genetics, Great Ormond Street Hospital, London, United Kingdom; 13 Pharmacology & Therapeutics, University of Manitoba, Winnipeg, Manitoba, Canada; 14 Pediatrics & Child Health, University of Manitoba, Winnipeg, Manitoba, Canada; 15 Windows of Hope Genetic Information Centre, Holmes County, Ohio, United States of America; 16 Human Genetics and Genomic Medicine, Faculty of Medicine, University of Southampton, Southampton, United Kingdom; 17 Department of Genetics, King Faisal Specialist Hospital and Research Center, Riyadh, Saudi Arabia; 18 Department of Anatomy and Cell Biology, College of Medicine, Alfaisal University, Riyadh, Saudi Arabia; 19 Manitoba Institute of Child Health, Winnipeg, Manitoba, Canada; University of Oxford, UNITED KINGDOM

## Abstract

Orofacial clefting is amongst the most common of birth defects, with both genetic and environmental components. Although numerous studies have been undertaken to investigate the complexities of the genetic etiology of this heterogeneous condition, this factor remains incompletely understood. Here, we describe mutations in the *HYAL2* gene as a cause of syndromic orofacial clefting. *HYAL2*, encoding hyaluronidase 2, degrades extracellular hyaluronan, a critical component of the developing heart and palatal shelf matrix. Transfection assays demonstrated that the gene mutations destabilize the molecule, dramatically reducing HYAL2 protein levels. Consistent with the clinical presentation in affected individuals, investigations of *Hyal2*^-/-^ mice revealed craniofacial abnormalities, including submucosal cleft palate. In addition, cor triatriatum sinister and hearing loss, identified in a proportion of *Hyal2*^-/-^ mice, were also found as incompletely penetrant features in affected humans. Taken together our findings identify a new genetic cause of orofacial clefting in humans and mice, and define the first molecular cause of human cor triatriatum sinister, illustrating the fundamental importance of HYAL2 and hyaluronan turnover for normal human and mouse development.

## Author Summary

Cleft lip and palate (CLP) is one of the most common congenital anomalies worldwide. Most of the time the cause is unknown. Affected individuals have multiple problems including difficulties with feeding and speech and have to undergo several operations to correct the orofacial cleft. We describe mutations in an enzyme involved in the development of the palate and heart (Hyaluronidase 2, HYAL2) as the cause of a syndrome of CLP and a congenital heart anomaly (cor triatriatum sinister, CTS) in families from the Amish community and Northern Saudi Arabia of Arabic ethnicity. Mice who lack this enzyme show similar features. HYAL2 is involved in the metabolism of hyaluronic acid, which is important in many tissues of the body. By illustrating a lack of HYAL2 causes CLP and CTS we have demonstrated the importance of HYAL2 for the normal development of the palate and heart, improving our understanding of the complex mechanisms involved in the pathogenesis of CLP and CTS which may ultimately contribute to the development of novel therapies to treat these congenital anomalies.

## Introduction

Orofacial clefts, which include cleft lip (CL) with or without cleft palate (CLP) and cleft palate alone (CP), collectively referred to as CL/P, are amongst the most common birth defects in all populations worldwide with many syndromic and non-syndromic forms described. The majority of cases are of unknown molecular cause [1]. The average incidence of CL/P is one in 700 newborns, with wide variability across racial and ethnic groups and socioeconomic status [2, 3]. The frequency of CL/P also differs by gender and laterality, there being a 2:1 male to female ratio for CLP, and a 1:2 male to female ratio for CP [2, 4]. Orofacial clefts arise from a failure of the intricate molecular and cellular processes that regulate bilateral fusion of the future lip and palate during craniofacial development [1]. They are clinically categorized dependent upon the absence (non-syndromic CL/P; 70% of cases) or presence (syndromic CL/P; 30% of cases) of additional congenital anomalies [5, 6]. For non-syndromic CL/P, 17 genetic risk loci have been defined and replicated with genome wide association studies worldwide [7–11]. As well as environmental influences, more recent evidence implicates non-coding or regulatory genomic regions in non-syndromic CL/P [12–14], perhaps explaining why re-sequencing of candidate genes or exome sequencing strategies rarely identify mutations in these cases [15]. For syndromic forms of CL/P, >300 different syndromes have been described in which CL/P is a prominent feature [16]. Congenital heart disease (CHD) is commonly associated with syndromic CL/P with a reported incidence of 13%-27% [15, 17–22]. Cor triatriatum is a rare congenital cardiac anomaly reported in 0.1–0.4% of individuals with congenital heart disease [23, 24]. It is characterized by division of either the left (cor triatriatum sinister) or less commonly the right atrium (cor triatriatum dexter) [25, 26]. To date, no molecular cause has been identified for cor triatriatum in humans. In the current study, we identify mutations in the gene *HYAL2* as a new genetic cause of orofacial clefting in humans and mice and describe the first molecular basis for cor triatriatum sinister in humans. Our findings illustrate the fundamental importance of hyaluronan (HA) turnover for normal human and mouse development.

## Results

### Clinical findings

#### Amish pedigree

**Fig 1:**
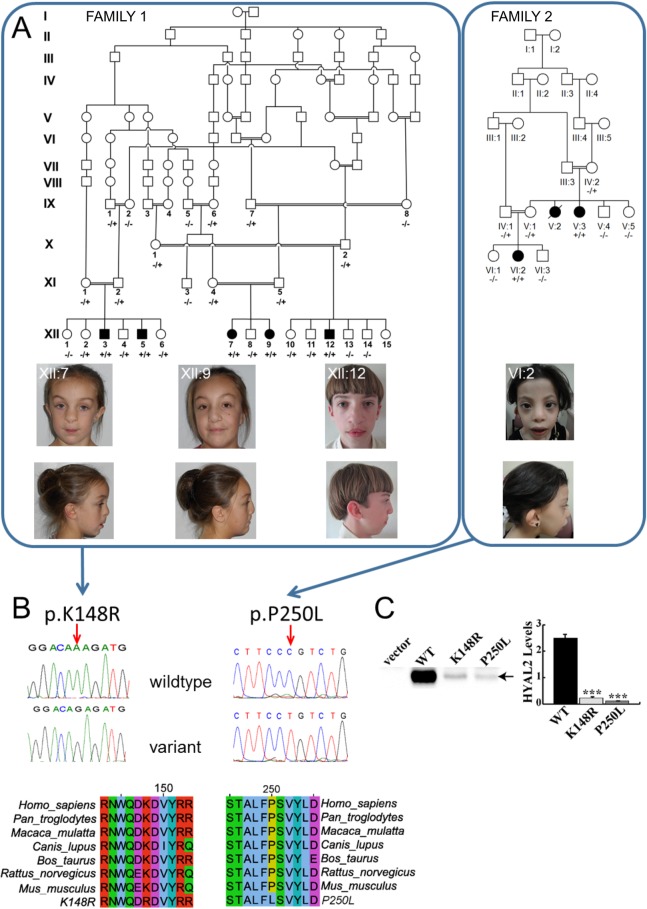
Pedigrees, clinical features of individuals homozygous for *HYAL2* mutation and identified *HYAL2* mutations, expression of wild type (WT), K148R-HYAL2 and P250L-HYAL2. (A) Pedigree diagrams and facial phenotype of individuals (Amish Family 1: XII:7; XII:9; XII:12 and Saudi Family 2: VI:2) with HYAL2 deficiency. Note the craniofacial similarities including frontal bossing, hypertelorism, widened nasal bridge, flattened broad nasal tip and cupped ears/overfolding of the superior helices. Consent for publication of these photographs was obtained (B) Electropherograms showing the identified c.443A>G & c.749C>T mutations and conservation of protein sequence across species.(C) Expression of wild type (WT), K148R and P250L-HYAL2. Western blots were performed on lysates prepared from MEFs deficient in HYAL2 that were transfected with empty vector, pCMV6-HYAL2, pCMV6-HYAL2K148R, or pCMV6-HYAL2P250L. An arrow indicates HYAL2. HYAL2 levels shown in the graph were quantified by imaging the chemiluminescent signal using a BioRad ChemiDoc. The columns represent the average level (x 10^6^ light units) of HYAL2 ± SEM (n = 4). Significance was determined using the student’s T test. *** indicates p<0.0001.

Five Amish individuals aged 4–16 years affected by a novel syndromic form of CLP were identified from a single extended pedigree consisting of three interlinking kindreds within the Amish community (Fig 1A). Bilateral or unilateral CLP and consistent craniofacial dysmorphism are the principal features of the condition. Additional clinical findings include congenital cardiac anomalies, pectus excavatum, single palmar creases, hearing loss that was predominantly conductive in nature, myopia and other ocular abnormalities including cataract and staphyloma. 2–3 soft tissue syndactyly of the toes was found on examination of the Amish affected individuals, but was also seen in unaffected members of the family. Craniofacial features included frontal bossing, hypertelorism, widened and flattened nasal bridge and tip, cupped ears/thickened helices and micrognathia. Cardiac anomalies observed were variable but interestingly included non-obstructive cor triatriatum sinister (dividing the pulmonary venous confluence from the body of the left atrium with an anterior fenestration in the membrane).

#### Saudi Arabian pedigree

Two children aged 7 and 12 years from an extended consanguineous Arab family from Northern Saudi Arabia were previously identified [27]. On comparison with the Amish family, significant similarity in the clinical features was noted. Most notably, the presence of bilateral cleft lip and palate in one affected girl and a similar facial appearance with frontal bossing, hypertelorism, micrognathia, and a broad flat nasal bridge. Both girls also had bilateral single palmer creases, lens opacities, staphyloma and were myopic. Echocardiography had only been carried out on the youngest affected girl and revealed an abnormal mitral valve with accessory tissue and a small restrictive perimembranous ventricular septal defect covered with tricuspid aneurysmal tissue. The older affected girl had pectus excavatum but cardiovascular examination was otherwise normal.

The clinical features of the affected individuals are summarized in Table 1 and shown in Fig 1A. Whereas it is normal for mice to have a left sided superior vena cava, there is regression of the left superior cardinal vein caudal to the innominate vein during normal human development and only the failure of this regression leads to the persistence of a left sided superior vena cava (PLSVC). A PLSVC represents the most common congenital thoracic venous anomaly with a prevalence of 0.3–0.5% in the general population [28]. In 80–90% of individuals, the PLSVC drains into the right atrium via the coronary sinus, which leads to a dilated coronary sinus, but usually has no hemodynamic consequence [29].

**Table 1:** Clinical findings of individuals homozygous for *HYAL2* mutations

	FAMILY 1					FAMILY 2	
	XII:3	XII:5	XII:7	XII:9	XII:12	V:3	VI:2
**GENDER**	Male	Male	Female	Female	Male	Female	Female
***HYAL2* MUTATION**	c.443A>G	c.443A>G	c.443A>G	c.443A>G	c.443A>G	c.749C>T	c.749C>T
**AGE (YRS)**	12.3	15.5	4.3	9.9	12.7	7.1	12.3
**GROWTH**							
***Height (SDS)***	-0.54	0.45	-1.07	0.29	-0.4	-2.87	-2.87
***Head circumference (SDS)***	-0.67	-0.22	-0.28	-0.49	-1.6	-3.13	-2.59
**SKELETON**							
***Pectus excavatum***	-	✓	✓	-	✓	-	✓
**CRANIOFACIAL FEATURES**							
***Cleft lip and palate***	Bilateral CLP	RT unilateral CLP	Bilateral CLP	Bilateral CLP	Bilateral CLP	None	Bilateral CLP
***Frontal bossing***	Not known	Not known	✓	-	✓	-	-
***Hypertelorism***			✓	✓	✓	✓	✓
***Micrognathia***			✓	✓	✓	✓	✓
***Broad flat nasal bridge***			✓	✓	✓	✓	✓
***Ears***			-	Over folded superior helices	-	Cupped ears	Cupped ears
**HEART**							
***Congenital cardiac malformations***	LT cor triatriatum	Dilated coronary sinus consistent with persistent LSVC	Normal echocardiogram	Dilated coronary sinus consistent with persistent LSVC	Normal echocardiogram	Abnormal MV with accessory tissue	Normal echocardiogram
						Small restrictive perimembranous VSD covered with tricuspid aneurysmal tissue	
							
***Other cardiac abnormalities***		Thickened AV		Dilated LT and RT atria		Mild TR	
*** ***		Restricted RT aortic leaflet				Lt-Rt shunt	
*** ***		Mild AR				Mildly dilated LV	
**EYES**							
***Visual acuity***	Glasses since age 2	Glasses since age 6	Normal	Glasses since age 9	Glasses since age 9	Glasses since age 3	Glasses since age 4
** **	RT myopia -8.00	RT myopia -1.50			RT myopia -2.50	RT myopia -16.75	RT myopia -9.75
** **	LT hyperopia +3.25	LT hyperopia +1.75			LT myopia -2.50	LT myopia -15.00	LT myopia -16.00
** **							
***Other ocular abnormalities***	Rt staphyloma					Cortical lens opacities and staphyloma with small disc	Corneal opacity
	Myopic macular degeneration	-	-	-	-		High myopia
	Posterior subcapsular polar cataract/ Mittendorf dot						Flat retina
							Staphyloma and LT retinal scar
**HEARING**							
***Conductive hearing loss***	RT moderate	-	Bilateral mild	-	LT moderate	RT Mild	-
*** ***	Postlingual				Prelingual		
***Sensorineural hearing loss***	-	Bilateral severe to profound	-	-	-	-	-
*** ***		Prelingual					
		LT cochlear implant					
**NORMAL PSYCHOMOTOR DEVELOPMENT**	✓	✓	✓	✓	✓	✓	✓
**HANDS AND FEET**	Not known	Not known	Single palmar crease - LT hand		Bilateral single palmar creases	Bilateral single palmar creases	Bilateral single palmar creases
** **			Bilateral 2-3 and 3-4 skin syndactyly of toes	Bilateral 2-3 and 3-4 skin syndactyly of toes	Bilateral 2-3 skin syndactyly of toes	Broad thumbs	
** **						Acromelia	

Abbreviations; SDS, standard deviation scores; (✓), indicates presence of a feature in an affected subject; (-), indicates presence of a feature in an affected subject); CLP, Cleft lip and palate; RT, Right; LT, Left; AV, Aortic valve; AR, Aortic regurgitation; LSVC, Left superior vena cava; LV, Left ventricle; VSD, Ventricular septal defect; TR, Tricuspid regurgitation; LV, Left ventricle

Height, weight and OFC Z-scores were calculated using a Microsoft Excel add-in to access growth references based on the LMS method [31] using a reference European population [32]

### Genetic studies

In order to map the chromosomal location of the disease gene we undertook a genome-wide SNP study in affected members of Family 1 assuming that a recessive founder mutation was responsible. This identified a single notable shared autozygous region of 10.18Mb on chromosome 3p21.31 (rs6441961-rs2201057; chr3:46310893–56499374 [hg38]) as likely to correspond to the disease locus. In support of this, multipoint linkage analysis with Simwalk2 [30] assuming autosomal recessive inheritance, full penetrance and a disease allele frequency of 0.0001, achieved a LOD_MAX_ = 10.37 corresponding to the autozygous interval. To identify the causative mutation, whole exome sequence analysis of a single affected individual (XII:3) was undertaken (Otogenetics Corporation). After filtering for call quality, potential pathogenicity, population frequency and localization within the candidate interval, we identified only a single likely deleterious variant located in the *HYAL2* gene (*HYAL2* chr3:g.50320047T>C [hg38]; NM_003773.4:c.443A>G; p.K148R; PolyPhen = probably damaging (1); SIFT = deleterious (0.03) MutationTaster = disease causing (probability 0.999997) Fig 1B). The variant was validated by Sanger dideoxy sequencing, which was also used to confirm co-segregation of the variant within the extended Amish pedigree (Fig 1A). Seven heterozygous carriers of the c.443A>G variant were identified in 532 Amish control chromosomes examined, indicating an allele frequency of approximately 0.013 in the population. The variant was not listed in the Exome Variant Server (NHLBI GO Exome Sequencing Project (ESP), Seattle, WA; http://evs.gs.washington.edu/EVS/), 1000 Genomes browser (http://browser.1000genomes.org/index.html) or the Exome Aggregation Consortium (ExAC) database (http://exacbroadinstitute.org/). Interestingly, *HYAL2* had recently been identified as a candidate gene as a cause of short stature and facial dysmorphism in a study of multiplex consanguineous families [27]. This study undertook genetic studies in 33 heterogeneous families with dysmorphic and other systemic clinical manifestations, and identified a sequence alteration in *HYAL2* as a possible cause of the clinical features in a single family comprising two affected individuals (Fig 1A, Family 2). Although inconclusive, when considered together with the genetic, clinical and functional data from the Amish study, this permits the robust genetic definition and clinical description of a new form of syndromic CLP. Genetic studies in Family 2 comprised whole genome SNP mapping which identified a single autozygous interval of 13.49Mb on chromosome 3p21 (rs7650433-rs9811393; chr3:40857673–54345775 [hg38]) shared by two affected family members, in which a missense *HYAL2* variant (chr3:g.50319741G>A [hg38]; NM_003773.4:c.749C>T; p.P250L in Family 2; Fig 1A and 1B), absent in 817 ethnically matched individuals, was identified as the candidate genetic cause. This variant (rs781999115) was not listed in the Exome Variant Server or the 1000 Genomes browser but two carriers were reported on the ExAC database. The genomic autozygous region (chr3:46310893–54379802 [hg38]) common to both family cohorts is 8.0Mb and predicted to contain 270 RefSeq alignment-supported transcripts (S1 Fig).The p.K148R and p.P250L substitutions decrease protein stability.

*HYAL2* encodes hyaluronidase 2, a membrane localized protein [33] with weak activity toward HA [34], which is an extracellular matrix glycosaminoglycan that is abundant during development. To assess the impact of p.K148R and p.P250L on the HYAL2 protein, the mutations were each introduced into a human HYAL2 expression construct and transiently transfected into MEFs deficient in HYAL2. Western blot analysis revealed a profound effect of the p.K148R and p.P250L mutations on protein levels, resulting in 11 and 20 fold reductions, respectively, compared to wild type (Fig 1C).

### Evaluation of craniofacial features and palate in *Hyal2*^*-/-*^ mice

Consistent with the facial dysmorphism in the human affected individuals, *Hyal2*^*-/-*^ mice exhibited a short broad nose, increased interorbitary space, and Wormian bones in the interfrontal suture [35]. To determine if the CL/P seen in the human affected individuals was also present in *Hyal2*^*-/-*^ mice, we examined the time of pre-weaning lethality described to affect 2/3 of knockouts [35, 36]. Among 201 progeny of *Hyal2*^*+/-*^ intercrosses followed from birth to weaning, 16 died at postnatal day (P)1, 16 died between P2-P9, and 16 *Hyal2*^*-/-*^ mice survived (S1 Table). Assuming all pups that died were *Hyal2*^*-/-*^, 48 of 201 progeny (24%) would be *Hyal2*^*-/-*^, approximating the Mendelian frequency for an autosomal recessive condition. We also studied ninety E18.5 embryos from *Hyal2*^*+/-*^ intercrosses and found two *Hyal2*^*-/-*^ and one *Hyal2*^*+/-*^ embryo(s) that had died at approximately E15.5. Taking these intrauterine deaths into account, the proportion of *Hyal2*^*+/+*^ mice still exceeded that of *Hyal2*^*-/-*^ mice (30% vs 24%, S1 Table). Although this difference did not reach significance, it suggested reduced survival of *Hyal2*^*-/-*^ and *Hyal2*^*+/-*^ embryos during early gestation.

**Fig 2:**
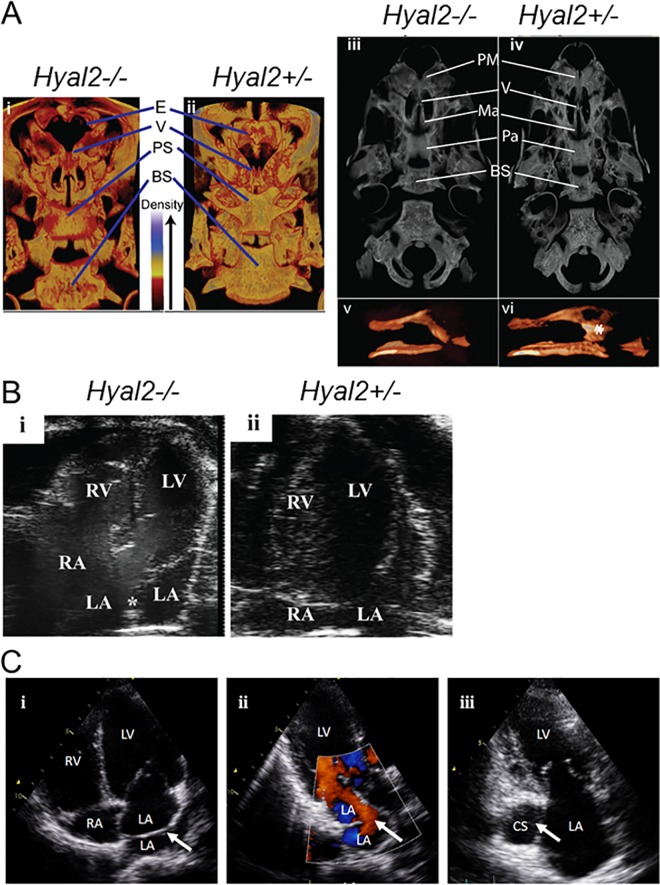
**Micro-CT images of the skulls of *Hyal2***^***-/-***^ **and *Hyal2***^***+/-***^ **mice, transthoracic echocardiography images of Amish individuals homozygous for *HYAL2* mutation c.443A>G, cardiac analysis in *Hyal2***^***-/-***^ **and *Hyal2***^***+/+***^ **mice** (A) 3D Images of *Hyal2*^*-/-*^ mice that died at P1, and the control (*Hyal2*^*+/-*^*)* which was sacrificed at P2, were created using CTvox software. All images were scanned and reconstructed using identical parameters and the dynamic range for the grey scale was held constant (i and ii). Dorsal views from the rostral end of the skull were prepared using a transfer function that colorized the grey scale such that areas of the bone with lower density are red (see density scale in image). (iii and iv). Ventral grey scale views of the skulls without the lower jaw. (v and vi). Space filled images of the vomer. * indicates the vomer head. Similar findings were evident in three additional *Hyal2*^*-/-*^ mice found dead at P1. These images are not to scale. E, ethmoid; V, vomer; PS, presphenoid; BS, basisphenoid; PM, premaxilla; MA, maxilla; PA, palantine. (B) (i & ii) Apical 4-chamber image of the heart is shown with right atrium (RA), left atrium (LA), right ventricle (RV), and left ventricle (LV) labelled. Presence of membranous tissue (*) in the LA of *Hyal2*^*-/-*^ mouse representing cor triatriatum sinister. (C) (i) Apical four chamber view of Amish individual XII:3: arrow indicates the membrane across the left atrium dividing the pulmonary venous confluence from the body of the left atrium (cor triatriatum sinister). (ii) Apical three chamber view with color. Doppler of individual XII:3: arrow indicates the anterior fenestration in the membrane dividing the left atrium, the laminar flow through the fenestration implies the non-obstructive nature of the membrane. (iii) Apical two chamber view of individual XII:9: arrow indicates the dilated coronary sinus indicative of a persistent left superior vena cava; RV: right ventricle, RA: right atrium, LV: left ventricle, LA: left atrium, CS: coronary sinus.

CL/P was a likely cause of death in *Hyal2*^*-/-*^ mice at P1 and possibly between P2-P9, whereas heart defects were the more probable cause at E15.5. Although most deceased pups were cannibalized before their genotype could be verified, four found dead at P1, without milk in their stomachs, were identified as *Hyal2*^*-/-*^. Micro-CT studies of these pups showed an underdeveloped and underossified viscerocranium compared to littermate controls (Fig 2Ai-ii). Several central palate bones were underdeveloped and the vomer did not fuse centrally or form a head that articulated with the maxilla (Fig 2Aiii-vi). The ethmoid bone was nearly absent (Fig 2Ai-ii), or severely reduced in size (S2C and S2D Fig). This underdevelopment of the viscerocranium indicated a defect in intramembranous ossification that was more prominent in the anterior midline and varied in severity among affected skulls (S2A–S2D Fig).

Characterization of the palates of *Hyal2*^*-/-*^ E18.5–19.5 embryos under a dissecting microscope revealed partial clefts and/or shortening of the secondary palate as well as abnormally formed rugae in most *Hyal2*^*-/-*^ mice and a small number of *Hyal2*^*+/-*^ mice (S3A–S3D Fig). Micro-CT confirmed reduced ossification and underdevelopment of the viscerocranial bones, particularly the vomer, consistent with submucosal cleft palate (SMCP) in 15 of 18 (83%; 95% CI 58–96%) *Hyal2*^*-/-*^, 3 of 54 (5.6%; 95% CI 1–16%) *Hyal2*^*+/-*^, and 0 of 23 (0%; 95% CI 0–18%) *Hyal2*^*+/+*^mice. Palatal malformation and clefting are likely to contribute significantly to the pre-weaning lethality in *Hyal2*^*-/-*^ mice, indicating further phenotypical overlap with the human condition.

Histological studies of P1 mice confirmed that the viscerocranial bones of all *Hyal2*^*-/-*^ mice were underdeveloped (S3E and S3F Fig, and arrows in N, O). Coronal sections showed the failed fusion between the epithelial surface of the vomeronasal organ and the dorsal side of the palate shelf (* in S3L and S3N Fig), leaving an average gap of 472 ± 50μm (n = 3) in *Hyal2*^*-/-*^ palates that was absent in control palates (S3M and S3O Fig). HA levels were clearly increased in the *Hyal2*^*-/-*^ tissues (S3J and S3N vs S3K and S3O Fig). Excess mesenchymal cells and their surrounding matrix, was also evident, particularly in the anterior head (* in S3H and S3I Fig).

### Evaluation of the heart in *Hyal2*^*-/-*^ mice

Valvular thickening and atrial dilatation are found in all *Hyal2*^*-/-*^ mice, although the severity of the phenotype varies [36]. Cor triatriatum sinister has been detected in 50% of *Hyal2*^*-/-*^ mice ([37] and Fig 2B). It is conceivable that deaths in the progeny of *Hyal2*^*+/-*^ intercrosses during embryogenesis and between P2-P9 observed are due to more severe (congenital) cardiac anomalies in *Hyal2*^*-/-*^ mice. The cardiac findings in the five individuals with the HYAL2 K148R variant display significant similarities including one individual with cor triatriatum sinister and two further individuals with dilated coronary sinuses indicative of a PLSVC (see Table 1 and Fig 2C). This provides convincing evidence of the importance of HYAL2 for normal heart development in humans and mice [36, 37] and identified mutation of *HYAL2* as the first described molecular mechanism for cor triatriatum sinister in humans.

### Evaluation of hearing in the *Hyal2*^*-/-*^ mice

Although conductive hearing loss was seen in most of the affected human subjects in this study, one affected individual was found to have profound sensorineural hearing loss. To assess hearing in *Hyal2*^*-/-*^ mice (8–12 weeks of age), the auditory brainstem response (ABR) was evaluated (S4 Fig). *Hyal2*^*-/-*^ mice exhibited a significantly higher threshold at all frequencies tested, (mean +/- 95% confidence interval for *Hyal2*^*-/-*^ and *Hyal2*^*+/+*^ mice were 17.6 +/-7.16 and 5.00 +/-3.88dB respectively; Mann-Whitney U-test U = 1433, p<0.001), indicating hearing loss in 100% of *Hyal2*^*-/-*^ mice. The ABR test parameters did not differentiate conductive and sensorineural hearing loss, but because these were performed on adult mice, severe craniofacial defects were excluded, although minor defects may be present. The presence of hearing loss in mice without severe palate defects suggests more studies are needed to determine whether HYAL2 has a direct role in the development and/or maintenance of normal hearing.

### HA accumulation in *Hyal2*^*-/-*^ deficiency

Previous studies of *Hyal2*^*-/-*^ mice with a complete absence of HYAL2 activity have demonstrated levels of circulating HA that are 19 fold higher than that of control mice at 12 weeks of age and accumulation of HA in the heart and lung [36] as well as other tissues [37]. In the current study, the HA levels were clearly elevated throughout the nasopharynx and in the palate (S3J, S3K, S3N and S3O Fig). It remains unclear to which degree this reflects species differences, but this finding is consistent with our transfection studies which show residual HYAL2 is present. This level of HYAL2 may be sufficient for constitutive turnover, but inadequate during development when rapid turnover of high levels of HA in the provisional matrix is required.

## Discussion

The molecular basis of human CLP is incompletely understood, despite its frequent occurrence and associated morbidity. Our findings demonstrate that a deficiency of the HA-degrading enzyme HYAL2 is a novel cause of syndromic CLP in humans and SMCP in mice, and define the first molecular explanation for cor triatriatum sinister in humans. All but one affected individual exhibited orofacial clefting, and craniofacial dysmorphism was a consistent finding. Myopia and other ocular abnormalities, pectus excavatum, single palmar creases, cor triatriatum sinister, a persistent left superior vena cava and other cardiac features were variably present. Our studies show that *Hyal2*^*-/-*^ mice universally exhibit facial dysmorphism, an underdevelopment of the viscerocranium and cardiac valve thickening as well as variably penetrant hearing loss, partial or SMCP (83%), cor triatriatum sinister (50%) and atrial dilatation (50%). The significant parity between the human and mouse phenotypes provides strong evidence that HYAL2-deficiency is the cause of these developmental abnormalities.

Our data indicate that the differences between the human and mouse phenotypes may be largely explained by the complete absence of HYAL2 in *Hyal2*^*-/-*^ mice, while the destabilizing and deactivating p.K148R and p.P250L HYAL2 substitutions identified in human affected individuals lead primarily to profound reductions in HYAL2 levels, which may still permit limited residual functionality. In surviving *Hyal2*^*-/-*^ mice progressive accumulation of HA in the circulation, pulmonary fibrosis, and cardiac dysfunction lead to premature heart failure [36], while the absence of elevated circulating HA in the affected Amish individuals may partially or completely protect them from these cardiopulmonary complications. The residual activity associated with the amino acid substitutions in human subjects likely supports ongoing constitutive turnover of HA, but is insufficient for the rapid and regulated turnover required during development. Further support for this is provided by our study of *Hyal2*^*+/-*^ mice where a small number showed developmental abnormalities in the absence of elevated circulating HA [36]. However, this may be an overly simplistic interpretation as circulating HA levels are approximately 10–20 fold higher in the mouse, despite similar rates of turnover in mice and humans [38]. The identification of additional HYAL2-deficient individuals would undoubtedly provide a more complete understanding of the phenotypic spectrum associated with HYAL2-deficiency.

We identified SMCP as a likely cause of early pre-weaning lethality in *Hyal2*^*-/-*^ mice. The underdeveloped viscerocranium likely weakens the palate, and in combination with the facial dysmorphism, presents significant feeding difficulties for *Hyal2*^*-/-*^ suckling pups. Neurological abnormalities could also contribute to death, as the cribriform plate of the ethmoid is important in supporting the olfactory bulb and in preventing the leakage of cerebrospinal fluid into the nasal cavity. The bones of the viscerocranium have not yet been examined in affected individuals with the *HYAL2* variant, but defects of the vomer are common in affected individuals with CLP [39]. The identification of developmental abnormalities in a small number of heterozygous (*Hyal2*^*+/-*^) mice suggests that haploinsufficiency of *HYAL2* may also pose the risk of a similar outcome in humans. Consistent with the notion that altered HYAL2 levels may affect palatal development, decreased HA has previously been associated with increased risk for CP in the *Tbx1*^*-/-*^ mouse [40], whereas increased HA has been associated with CP in *Sim2*^*-/-*^ mice [41]. Thus, *HYAL2* variants may be considered as candidates, even in the heterozygous form, to confer risk for these developmental abnormalities. However screening a cohort of non-syndromic CL/P individuals, provided little evidence for a major role for *HYAL2* in this cohort (personal communication- P. Stanier). The cohort consisted of 380 individuals (176 with unilateral or bilateral cleft lip and palate, 38 with cleft lip only, 121 with cleft palate only and 46 with submucous cleft palate) of a mixture of ethnicities the majority of which (72%) were of white European origin recruited from the North East Thames region of the UK. Approximately 22% of cases were familial with affected first or second-degree relatives. While this study cohort is too small to draw definitive genotype-phenotype conclusions, this finding remains consistent with *HYAL2* mutation being likely only associated with a rare, autosomal recessive syndrome.

Eloquent studies in mice deficient in HA synthesis (*Has2*^*-/-*^) demonstrated that high molecular mass HA was essential for epithelial to mesenchymal transition (EMT) in the developing heart, whereas HA oligosaccharides inhibited EMT and promoted angiogenesis [42–44]. HYAL2 could modulate the size of HA to inhibit EMT and promote differentiation, in which case its deficiency would result in increased levels of high molecular mass HA, an overproduction of mesenchymal cells, and a decrease in differentiation. Indeed, the thickened cardiac valves and excess mesenchymal cells in some parts of the head of *Hyal2*^*-/-*^ mice, suggest that EMT is upregulated in *Hyal2*^*-/-*^ mice, and that HYAL2 normally plays a role in inhibiting this process. In histological sections, encompassing the nasopharynx and secondary palate, there is an abundance of cartilage but a deficiency in bone. The deficient ossification in the skull of *Hyal2*^*-/-*^ mice, and underdevelopment of the viscerocranium, suggests a failure in the development of osteoblasts, as has been seen in *Tbx22*^*-/-*^ mice [45].

In addition to the direct effects of HA on signaling pathways, it is possible that an overabundance of HA in *Hyal2*^*-/-*^ tissues leads to alterations in the gradients of morphogens or signal transduction factors, impacting development. Clearly further studies are needed to investigate this possibility. Treatment strategies for this developmental disorder deserve further research, as HA synthesis can be inhibited using 4-methylumelliferone, an FDA-approved drug in some parts of the world [46]. Further, commercial preparations of human hyaluronidase that function at a neutral pH could be used to remove extracellular HA; these enzymes are available for human use [47], and formulations that are stable in the circulation are under development [48]. With the *Hyal2*^*-/-*^ mouse model already available, testing of such strategies may enable the development of a pre-natal treatment for this malformation syndrome. Taken together, our findings highlight hyaluronidase enzymes as playing a vital role in both human and mouse development, and in particular have revealed a previously unrecognized pathway involved in the pathogenesis of orofacial clefting, myopia and cor triatriatum sinister in humans, which may ultimately be amenable to treatment therapies.

## Materials and Methods

### Ethics statement

Signed consent was obtained for all individuals and the study was approved by the institutional review board at the University of Arizona (reference 10-0050-01) and the Great Ormond Street Hospital and the Institute of Child Health Research Ethics Committee (REC No. 08H0713/46). Animals were euthanized by isoflurane overdose and embryos were collected for gross morphological or histological studies. All procedures were in compliance with the Canadian Council on Animal Care and followed a protocol approved by the University of Manitoba Animal Care Committee.

### Genetic studies

Blood samples for DNA extraction were obtained with informed consent from all participating family members (institutional review board-approved research protocols UoA:10-0050-10 and KFSHRC RAC#2080006). A genome-wide SNP screen was undertaken in the affected siblings (Family 1: XII:3; XIII:5; XII:7; XII:9; XII:12, and Family 2: V:3; VI:2) using Illumina HumanCytoSNP-12 v2.1 or Axiom SNP Chip arrays for autozygosity mapping of regions of >1Mb using AutoSNPa [49]. Whole exome sequencing of genomic DNA from Family 1 was performed at Otogenetics Corporation (Norcross, GA, USA) using the Agilent SureSelect Human All ExonV4 (51Mb) enrichment kit with a paired-end (2 × 100) protocol at a mean coverage of 30X. For Family 2, we used a TruSeq Exome Enrichment kit (Illumina, San Diego, CA) with samples prepared as an Illumina sequencing library enriched for the desired targets using the Illumina Exome Enrichment, with captured libraries sequenced using an Illumina HiSeq 2000 Sequencer. Sequence reads were aligned to the human genome reference sequence [hg19] and read alignment, variant calling, and annotation were performed by DNAnexus (DNAnexus Inc, Mountain View, CA). Intronic variants not predicted to affect splicing or regulation were excluded and SIFT [50], PolyPhen-2 [51], and MutationTaster [52] was used to predict the impact of any identified amino acid substitution on the protein structure and function and to predict and prioritize potential disease causing sequence alterations. The presence of the variants were confirmed in the transcript by bidirectional dideoxy Sanger sequencing performed on an ABI3730 XL capillary sequencer (Applied Biosystems), which was also used to confirm its co-segregation within the respective pedigrees.

### Experimental animals

*Hyal2*^*-/-*^ (null) mice and littermate controls (*Hyal2*^*+/-*^ or *Hyal2*^*+/+*^) were obtained from heterozygous intercrosses of mice maintained on an outbred (C129; CD1; C57BL/6) background as viable *Hyal2*^*-/-*^ mice have not been obtained on an inbred background [35]. Embryos were considered E0.5 the morning a vaginal plug was discovered. Genotyping was performed as described [36]. Animals were euthanized by isoflurane overdose and embryos were collected for gross morphological or histological studies. All procedures were in compliance with the Canadian Council on Animal Care and followed a protocol approved by the University of Manitoba Animal Care Committee.

### Animal imaging, hearing analysis and histological studies

Ultrasound imaging was performed on mice anesthetized with 2% isoflurane, using a Visual Sonics Vevo 2100 ultrasound equipped with a 40 MHz transducer. Micro-CT imaging was performed at 9μm resolution with a Skyscan 1176 micro-CT and images were reconstructed and analyzed using Bruker’s NRecon, Data Viewer, CT Analyzer, or CT Vox software. ABR testing was performed as described previously [53]. Skeletal staining of neonatal mice was as described in [54]. For histological studies, tissues were fixed in 1% cetylpyridinium chloride in 10% buffered formalin, decalcified if appropriate, imbedded in paraffin, and 5μm sections were prepared. Sections were stained with H & E for analysis of morphology. HA was detected using the HA binding protein (HABP) following established protocols [36].

### HA and hyaluronidase assays

HA levels were determined using a DuoSet enzyme linked immunosorbent assay (ELISA) development kit (R & D Systems).

### Transient transfection and western blot analysis

Transfections of HYAL2-expressing vectors were into mouse embryonic fibroblasts (MEFs) derived from a *Hyal2*^*-/-*^ embryo. Cells and culture medium were collected 48 hours post-transfection and the β-galactosidase activity was determined using O-nitrophenol-β-D-galactopyranoside as a substrate [55]. Protein concentrations in the lysates were determined using a BioRad protein assay kit based on the Bradford method, and β-galactosidase levels were used to normalize for transfection efficiency in all assays. Western blot analysis was as described previously, but using polyclonal anti-HYAL2 (Proteintech) [56].

## Supporting Information

S1 Table Genotypes and survival of progeny of *Hyal2^+/-^* intercrosses. (DOCX)

S1 Fig Visualization of regions of genetic homozygosity. Pictorial representation of the SNP genotype data encompassing the chromosome 3 homozygous regions in affected individuals. The locus containing the pathogenic variant is demarcated by SNPs (rs6441961 and rs9811393 8.03Mb; 270 genes).
(TIF)

S2 Fig Visualization of the skulls of *Hyal2^-/-^* and *Hyal2^+/-^* mice. The skulls of *Hyal2*^*-/-*^ mice that died at P1, and of a *Hyal2*^*+/-*^ mouse that was sacrificed at P2, were visualized by skeletal staining (A,B) or micro-CT imaging (C,D). (A and B) Photographs of the anterior region of the viscerocranium after removal of the skull. The ethmoid (E) bone is severely underdeveloped in the *Hyal2*^*-/-*^ mouse compared to the *Hyal2*^*+/-*^ mouse. For micro-CT imaging, the scanning and reconstruction parameters, including the dynamic range of the grey scale, were the same for all images. (C and D) 3D micro-CT grey scale images of the ethmoid bone that is almost absent in the *Hyal2*^*-/-*^ mouse, but well developed in the *Hyal2*^*+/-*^ control. E-ethmoid bone.
(TIF)

S3 Fig Analysis of the palates of *Hyal2^-/-^*, *Hyal2^+/-^*, and *Hyal2^+/+^* mice. (A-D) Photographs were taken of the ventral surface of the head of E18.5 embryos after removing the bottom mandible. Arrows indicate partial cleft palates indicative of submucosal cleft palate. (E—G) Histological analysis of *Hyal2*^*-/-*^ and *Hyal2*^*+/+*^ P1 heads. Sagittal sections were stained with H & E. The reduction in staining intensity indicates reduced ossification in *Hyal2*^*-/-*^ mice compared to controls. The schematic in (G) indicates the identity of the bones: PM, premaxilla; MA, maxilla; PA, palantine; PS, presphenoid; BS, basisphenoid (H-I). Coronal sections through the nasopharynx and secondary palate of E18.5 embryos were stained for morphological analysis with hematoxylin and eosin. Areas with excess mesenchymal cells in the *Hyal2*^*-/-*^ mouse are indicated with asterisks. (J and K) Hyaluronan (brown) detected in coronal sections of the nasopharynx. (L and M) H & E staining of the fusion between the vomeronasal organ and palate. An * indicates the failed fusion in the *Hyal2*^*-/-*^ mouse. (N and O) Hyaluronan (brown) detection in the region of the vomeronasal/palate fusion. An * indicates the failed fusion between the vomeronasal organ and primary palate. A black arrow indicates the underdeveloped vomer and a green arrow indicates the underdeveloped maxilla. All images are representative of a minimum of 3 sets of *Hyal2*^*-/-*^ and control mice.
(TIF)

S4 Fig Hearing analysis in *Hyal2^-/-^* and *Hyal2^+/+^* mice. Evaluation of hearing in *Hyal2*^*-/****-***^ and control (*Hyal2*^*+/+*^) mice was assessed by the ABR (auditory brain stem response) at varying frequencies and intensities These values are based on the average of 16 ears (8 mice) for each genotype The bars represent the mean threshold and the 95% confidence interval.
(TIF)
